# Supplementary material for: Neural network and layer-wise relevance propagation reveal how ice hockey protective equipment restricts players’ motion
Source: PLoS One. 2024 Oct 15;19(10):e0312268. doi: 10.1371/journal.pone.0312268 (PMC11478874; doi:10.1371/journal.pone.0312268)
Supplement: S1 Table — Ranges of motion are given in the full range of motion and split values in the two movement directions. The ratio was calculated by dividing the individual joint angles by the overall maximum range of motion (shoulder abduction/adduction) [28]. (PDF) [file pone.0312268.s001.pdf]

Joint	Movement	Range of motion	Ratio
Ankle	Flexion / Extension	80 (50 / 30)	0.36
	Abduction / Adduction	---	
	Internal / External Rotation	36 (20 / 16)	0.16
Knee	Flexion / Extension	160 (150 / 10)	0.73
	Abduction / Adduction	---	
	Internal / External Rotation	55 (40 / 15)	0.25
Hip	Flexion / Extension	150 (140 / 10)	0.68
	Abduction / Adduction	90 (50 / 40)	0.41
	Internal / External Rotation	95 (45 / 50)	0.43
Shoulder	Flexion / Extension	220 (180 / 40)	1
	Abduction / Adduction	210 (170 / 40)	0.95
	Internal / External Rotation	150 (90 / 60)	0.68
Elbow	Flexion / Extension	160 (150 / 10)	0.73
	Pronation / Supination	180 (90 / 90)	0.82
	Ulnar / Radial Deviation	---	
