# Supplementary figures and images for: Neural network and layer-wise relevance propagation reveal how ice hockey protective equipment restricts players’ motion
Source: PLoS One. 2024 Oct 15;19(10):e0312268. doi: 10.1371/journal.pone.0312268 (PMC11478874; doi:10.1371/journal.pone.0312268)

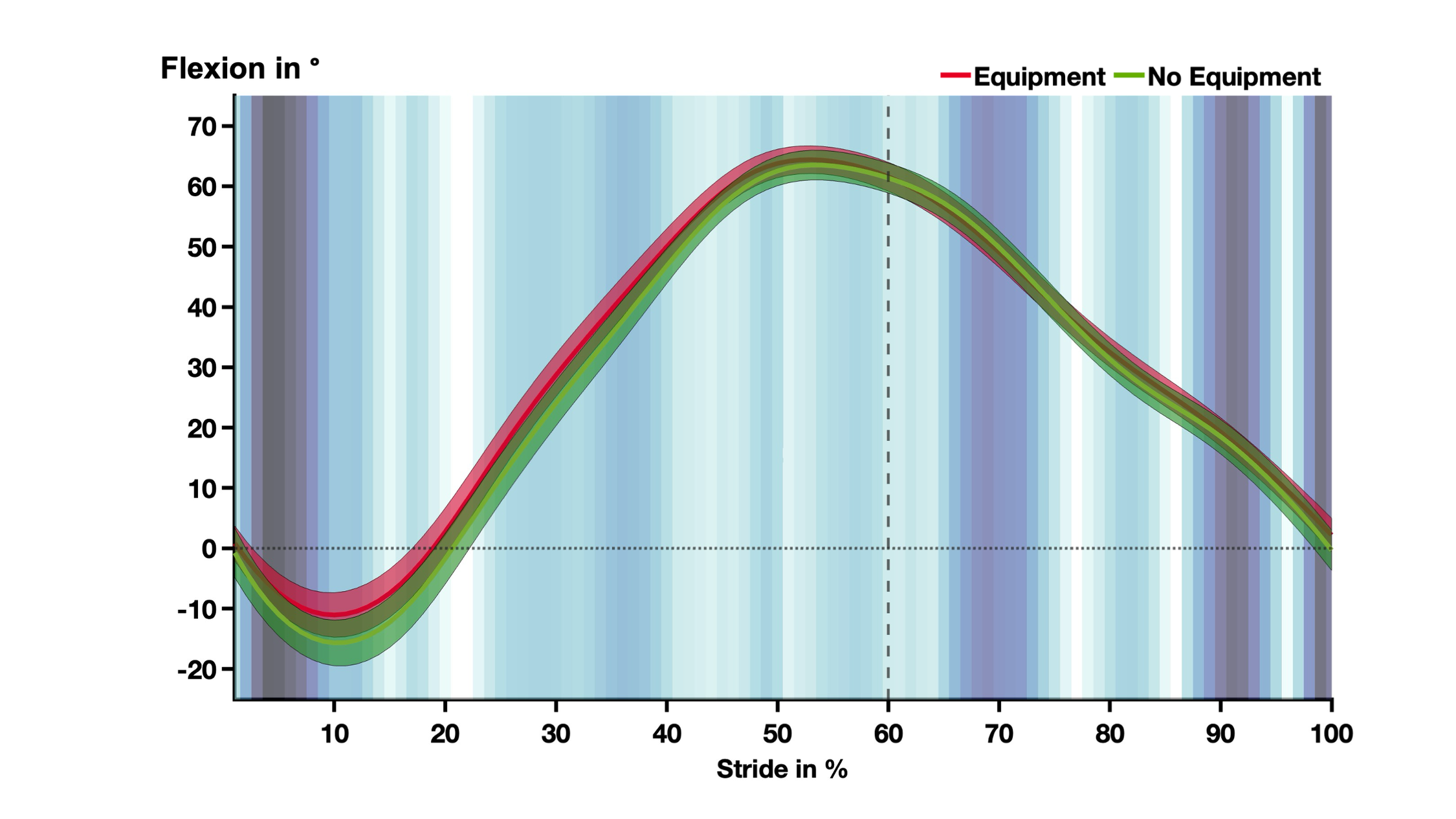

Supplement: S1 Fig — The joint trajectories are displayed with the mean (solid line) ± standard error (shaded area) for the two conditions, equipment in red and no equipment in green over one sprint stride cycle. The vertical line indicates the skate-off and therefore the transition from the gliding to the swing phase of the foot. The plots were overlayed with the relevance scores calculated for the shoulder flexion/extension trajectory. Dark colors indicate higher relevance and lighter colors indicate lower relevance. (TIF) [file pone.0312268.s002.tif]
