# Supplementary material for: Neural network and layer-wise relevance propagation reveal how ice hockey protective equipment restricts players’ motion
Source: PLoS One. 2024 Oct 15;19(10):e0312268. doi: 10.1371/journal.pone.0312268 (PMC11478874; doi:10.1371/journal.pone.0312268)
Supplement: S1 File — This archive contains all the underlying data (raw and processed) presented in this publication. (ZIP) [file pone.0312268.s003.zip › S1_File/Readme.docx]

This archive contains the data used to create the plots for the submitted manuscript.

The file **data.mat** is a matlab data file containing the underlying data. The following variables are included:

- **Performance:** Structure containing the performance data for each participant for the sprint (time in seconds), the linear crossover (time in seconds) and the shot (puck velocity in kilometers per hour). Each measurement was taken twice for each condition. The trial and the condition are indicated (E = with Protective Equipment, NE = Without Protective Equipment), e.g., Sprint NE2 was the second sprint trial without protective equipment.
- **RelevanceLinearCrossover:** Matrix (1x2400) containing the processed relevance scores obtained using Layer-wise relevance propagation for the linear crossover. The relevance scores were smoothed using the described filtering approach and the relevance scores of the correctly classified steps were averaged. The joint angles are concatenated containing 100 values per joint angle:
  - 1-100: Ankle Flexion / Extension (Following Leg)
  - 101-200: Ankle Internal / External Rotation (Following Leg)
  - 201-300: Knee Flexion / Extension (Following Leg)
  - 301-400: Knee Internal / External Rotation (Following Leg)
  - 401-500: Hip Flexion / Extension (Following Leg)
  - 501-600: Hip Abduction / Adduction (Following Leg)
  - 601-700: Hip Internal / External Rotation (Following Leg)
  - 701-800: Shoulder Flexion / Extension (Following Leg)
  - 801-900: Shoulder Abduction / Adduction (Following Leg)
  - 901-1000: Shoulder Internal / External Rotation (Following Leg)
  - 1001-1100: Elbow Flexion / Extension (Following Leg)
  - 1101-1200: Elbow Pronation / Supination (Following Leg)
  - 1201-1300: Ankle Flexion / Extension (Crossing Leg)
  - 1301-1400: Ankle Internal / External Rotation (Crossing Leg)
  - 1401-1500: Knee Flexion / Extension (Crossing Leg)
  - 1501-1600: Knee Internal / External Rotation (Crossing Leg)
  - 1601-1700: Hip Flexion / Extension (Crossing Leg)
  - 1701-1800: Hip Abduction / Adduction (Crossing Leg)
  - 1801-1900: Hip Internal / External Rotation (Crossing Leg)
  - 1901-2000: Shoulder Flexion / Extension (Crossing Leg)
  - 2001-2100: Shoulder Abduction / Adduction (Crossing Leg)
  - 2101-2200: Shoulder Internal / External Rotation (Crossing Leg)
  - 2201-2300: Elbow Flexion / Extension (Crossing Leg)
  - 2301-2400: Elbow Pronation / Supination (Crossing Leg)
- **RelevancePowerTurn:** Matrix (1x2400) containing the processed relevance scores obtained using Layer-wise relevance propagation for the power turn. The relevance scores were smoothed using the described filtering approach and the relevance scores of the correctly classified steps were averaged. The joint angles are concatenated containing 100 values per joint angle:
  - 1-100: Ankle Flexion / Extension (Inside)
  - 101-200: Ankle Internal / External Rotation (Inside)
  - 201-300: Knee Flexion / Extension (Inside)
  - 301-400: Knee Internal / External Rotation (Inside)
  - 401-500: Hip Flexion / Extension (Inside)
  - 501-600: Hip Abduction / Adduction (Inside)
  - 601-700: Hip Internal / External Rotation (Inside)
  - 701-800: Shoulder Flexion / Extension (Inside)
  - 801-900: Shoulder Abduction / Adduction (Inside)
  - 901-1000: Shoulder Internal / External Rotation (Inside)
  - 1001-1100: Elbow Flexion / Extension (Inside)
  - 1101-1200: Elbow Pronation / Supination (Inside)
  - 1201-1300: Ankle Flexion / Extension (Outside)
  - 1301-1400: Ankle Internal / External Rotation (Outside)
  - 1401-1500: Knee Flexion / Extension (Outside)
  - 1501-1600: Knee Internal / External Rotation (Outside)
  - 1601-1700: Hip Flexion / Extension (Outside)
  - 1701-1800: Hip Abduction / Adduction (Outside)
  - 1801-1900: Hip Internal / External Rotation (Outside)
  - 1901-2000: Shoulder Flexion / Extension (Outside)
  - 2001-2100: Shoulder Abduction / Adduction (Outside)
  - 2101-2200: Shoulder Internal / External Rotation (Outside)
  - 2201-2300: Elbow Flexion / Extension (Outside)
  - 2301-2400: Elbow Pronation / Supination (Outside)
- **RelevanceShooting:** Matrix (1x2400) containing the processed relevance scores obtained using Layer-wise relevance propagation for the shot. The relevance scores were smoothed using the described filtering approach and the relevance scores of the correctly classified steps were averaged. The joint angles are concatenated containing 100 values per joint angle:
  - 1-100: Ankle Flexion / Extension (Dominant side)
  - 101-200: Ankle Internal / External Rotation (Dominant side)
  - 201-300: Knee Flexion / Extension (Dominant side)
  - 301-400: Knee Internal / External Rotation (Dominant side)
  - 401-500: Hip Flexion / Extension (Dominant side)
  - 501-600: Hip Abduction / Adduction (Dominant side)
  - 601-700: Hip Internal / External Rotation (Dominant side)
  - 701-800: Shoulder Flexion / Extension (Dominant side)
  - 801-900: Shoulder Abduction / Adduction (Dominant side)
  - 901-1000: Shoulder Internal / External Rotation (Dominant side)
  - 1001-1100: Elbow Flexion / Extension (Dominant side)
  - 1101-1200: Elbow Pronation / Supination (Dominant side)
  - 1201-1300: Ankle Flexion / Extension (Non-dominant side)
  - 1301-1400: Ankle Internal / External Rotation (Non-dominant side)
  - 1401-1500: Knee Flexion / Extension (Non-dominant side)
  - 1501-1600: Knee Internal / External Rotation (Non-dominant side)
  - 1601-1700: Hip Flexion / Extension (Non-dominant side)
  - 1701-1800: Hip Abduction / Adduction (Non-dominant side)
  - 1801-1900: Hip Internal / External Rotation (Non-dominant side)
  - 1901-2000: Shoulder Flexion / Extension (Non-dominant side)
  - 2001-2100: Shoulder Abduction / Adduction (Non-dominant side)
  - 2101-2200: Shoulder Internal / External Rotation (Non-dominant side)
  - 2201-2300: Elbow Flexion / Extension (Non-dominant side)
  - 2301-2400: Elbow Pronation / Supination (Non-dominant side)
- **RelevanceSprint:** Matrix (1x1200) containing the processed relevance scores obtained using Layer-wise relevance propagation for the sprint. The relevance scores were smoothed using the described filtering approach and the relevance scores of the correctly classified steps were averaged. The joint angles are concatenated containing 100 values per joint angle:
  - 1-100: Ankle Flexion / Extension
  - 101-200: Ankle Internal / External Rotation
  - 201-300: Knee Flexion / Extension
  - 301-400: Knee Internal / External Rotation
  - 401-500: Hip Flexion / Extension
  - 501-600: Hip Abduction / Adduction
  - 601-700: Hip Internal / External Rotation
  - 701-800: Shoulder Flexion / Extension
  - 801-900: Shoulder Abduction / Adduction
  - 901-1000: Shoulder Internal / External Rotation
  - 1001-1100: Elbow Flexion / Extension
  - 1101-1200: Elbow Pronation / Supination
