# Supplementary material for: Neural network and layer-wise relevance propagation reveal how ice hockey protective equipment restricts players’ motion
Source: PLoS One. 2024 Oct 15;19(10):e0312268. doi: 10.1371/journal.pone.0312268 (PMC11478874; doi:10.1371/journal.pone.0312268)
Supplement: Attachment — Submitted filename: Response to Reviewers.pdf [file pone.0312268.s004.pdf]

Response to Reviewers

Dear Editor, dear Reviewers,

Our manuscript “Neural network and layer-wise relevance propagation reveal how ice hockey protective equipment restricts players' motion” was rejected based on the following decision:

> “Based on our evaluation of your manuscript and the information you provided, we do not feel that your submission meets our ethical requirements for human subjects research submissions. PLOS ONE requires that research meets all applicable standards for the ethics of experimentation and research integrity (http://journals.plos.org/plosone/s/human-subjects-research). We reserve the right to reject any submission that does not meet our internal ethical standards, which in some cases are more stringent than local ethical standards.
>
> We understand that you obtained ethical approval from your local ethics committee after you started recruiting participants. PLOS ONE does not accept retrospective ethical approval for human subjects research. As a result, we cannot consider the manuscript for publication at PLOS ONE.”

Accidentally, I sent the extension document instead of the original ethics approval, which got signed and approved (7-Jan-2022) before the start of recruitment (6-Apr-2022). Since this ethics approval was expiring after a year, we needed to renew it for some of the other projects covered by these ethics. I uploaded the correct version of the ethics approval including the Human Subjects Research Checklist. I sincerely apologize for mixing up the two documents. No changes were made to the manuscript.
